# Genome sequence and description of *Timonella senegalensis* gen. nov., sp. nov., a new member of the suborder *Micrococcinae*

**DOI:** 10.4056/sigs.3476977

**Published:** 2013-06-13

**Authors:** Ajay Kumar Mishra, Jean-Christophe Lagier, Catherine Robert, Didier Raoult, Pierre-Edouard Fournier

**Affiliations:** 1 Aix-Marseille Université, URMITE, Faculté de médecine, Marseille, France

**Keywords:** *Timonella senegalensis*, genome, culturomics, taxono-genomics

## Abstract

*Timonella senegalensis* strain JC301^T^ gen. nov., sp. nov. is the type strain of *T. senegalensis* gen. nov., sp. nov., a new species within the newly proposed genus *Timonella*. This bacterial strain was isolated from the fecal flora of a healthy Senegalese patient. In this report, we detail the features of this organism, together with the complete genome sequence and annotation. *Timonella senegalensis* strain JC301^T^ exhibits the highest 16S rRNA similarity (95%) with *Sanguibacter marinus*, the closest validly published bacterial species. The genome of *T. senegalensis* strain JC301^T^ is 3,010,102-bp long, with one chromosome and no plasmid. The genome contains 2,721 protein-coding genes and 72 RNA genes, including 5 rRNA genes. The genomic annotation revealed that *T. senegalensis* strain JC301^T^ possesses the complete complement of enzymes necessary for the *de novo* biosynthesis of amino acids and vitamins (except for riboflavin and biotin), as well as the enzymes involved in the metabolism of various carbon sources, chaperone genes, and genes involved in the regulation of polyphosphate and glycogen levels.

## Introduction

*Timonella senegalensis* strain JC301^T^ (= CSUR P167 = DSMZ 25696) is the type strain of *T. senegalensis* gen. nov., sp. nov. This bacterium is a Gram-positive, facultatively anaerobic, flagellated, indole-positive bacillus that was isolated from the feces of a healthy Senegalese patient in a study aiming at cultivating all bacterial species in human feces [1].

The current classification of prokaryotes, known as polyphasic taxonomy, relies on a combination of phenotypic and genotypic characteristics [2]. However, as more than 4,000 bacterial genomes have been sequenced [3] and the cost of genomic sequencing is decreasing, we recently proposed to integrate genomic information in the description of new bacterial species [4-22].

Here we present a summary classification and a set of features for *T. senegalensis* gen. nov., sp. nov. strain JC301^T^ (= CSUR P167 = DSMZ 25696) together with the description of the complete genomic sequencing and annotation. These characteristics support the circumscription of a novel genus, *Timonella* gen. nov. within the suborder *Micrococcineae*, with *Timonella senegalensis* gen. nov., sp. nov. as the type species.

The suborder *Micrococcineae* was created in 1997 [23] and currently comprises eighteen different families that mostly includes Gram-positive bacteria. Members of the suborder *Micrococcineae* are usually present in soil, water, terrestrial, marine environments, humans and animal intestinal microbiota.

## Classification and features

A stool sample was collected from a healthy 16-year-old male Senegalese volunteer patient living in Dielmo (rural village in the Guinean-Sudanian zone in Senegal), who was included in a research protocol. Written assent was obtained from this individual. No written consent was needed from his guardians for this study because he was older than 15 years old (in accordance with the previous project approved by the Ministry of Health of Senegal and the assembled village population and as published elsewhere [24]).

Both this study and the assent procedure were approved by the National Ethics Committee of Senegal (CNERS) and the Ethics Committee of the Institut Fédératif de Recherche IFR48, Faculty of Medicine, Marseille, France (agreement numbers 09-022 and 11-017). Several other new bacterial species were isolated from this specimen using various culture conditions, including the recently described *Aeromicrobium massiliense* sp. nov., *Alistipes senegalensis* sp. nov., *Alistipes timonensis* sp. nov., *Anaerococcus senegalensis* sp. nov., *Brevibacterium senegalense* sp. nov., *Cellulomonas massiliensis* sp. nov., *Clostridium senegalense* sp. nov., *Enterobacter massiliensis* sp. nov., *Herbaspirillum massiliense* sp. nov., *Kurthia massiliensis* sp. nov., *Paenibacillus senegalensis* sp. nov., *Peptoniphilus timonensis* sp. nov., and *Senegalemassilia anaerobia* gen. nov., sp. nov. [5-16, 18,19].

**Figure 1:**
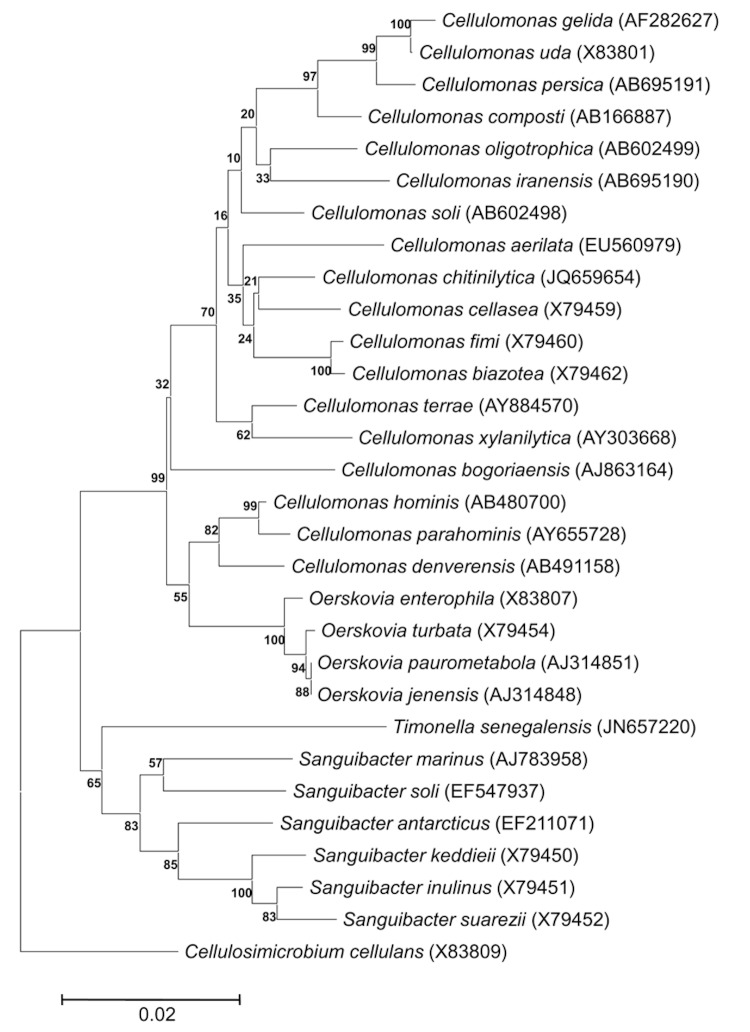
Phylogenetic tree highlighting the position of *Timonella senegalensis* strain JC301^T^ relative to several type species of suborder *Micrococcineae*. Genbank accession numbers are indicated in parentheses. Sequences were aligned using ClustalW, and phylogenetic inferences obtained using the maximum-likelihood method within the MEGA software package. Numbers at the nodes are bootstrap values obtained by repeating 500 times the analysis to generate a majority consensus tree. *Cellulosimicrobium cellulans* was used as an outgroup. The scale bar represents a 2% nucleotide sequence divergence.

The fecal specimen was preserved at -80°C after collection and sent to Marseille. Strain JC 301^T^ was isolated in June 2011 by cultivation on 5% sheep blood agar under anaerobic conditions at 37°C, after a 14-day preincubation in a blood culture bottle with sterile rumen sheep fluid. In the inferred phylogenetic tree (Figure 1), strain JC301^T^ fell into a large cluster containing the genera *Cellulomonas*, *Oerskovia* and *Sanguibacter*. In this cluster, strain JC301^T^ formed a distinct lineage. The 16S rRNA gene sequence identity between JC301^T^ and the type strains of related species (*Cellulomonas*, *Oerskovia* and *Sanguibacter*) of suborder *Micrococcineae* ranged from 92 to 95%. These values were lower than the threshold recommended by Schloss and Handelsman [25] to delineate a new genus without carrying out DNA-DNA hybridization, thus suggesting that strain JC301^T^ represents a novel genus. Based on the 16S rRNA phylogenetic evidence described above, we conclude that JC301^T^ represents a novel genus and species within the suborder *Micrococcineae* of the phylum *Actinobacteria* (see Table 1).

**Table 1:** Classification and general features of *Timonella senegalensis* strain JC301^T^ according to the MIGS recommendations [26]

**MIGS ID**	**Property**	**Term**	**Evidence code^a^**
		Domain *Bacteria*	TAS [27]
		Phylum *Actinobacteria*	TAS [28]
		Class *Actinobacteria*	TAS [23]
	Current classification	Order *Actinomycetales*	TAS [23,29-31]
		Family *Sanguibacteraceae*	TAS [32]
		Genus *Timonella*	IDA
		Species *Timonella senegalensis*	IDA
		Type strain JC301^T^	IDA
	Gram stain	positive	IDA
	Cell shape	short, irregular rods	IDA
	Motility	motile	IDA
	Sporulation	nonsporulating	IDA
	Temperature range	mesophile	IDA
	Optimum temperature	37°C	IDA
MIGS-6.3	Salinity	growth in BHI medium + 1% NaCl	IDA
MIGS-22	Oxygen requirement	primarily aerobic, facultatively anaerobic	IDA
	Carbon source	broad variety of sugars	IDA
	Energy source	carbohydrates	IDA
MIGS-6	Habitat	human gut	IDA
MIGS-15	Biotic relationship	free living	IDA
MIGS-14	Pathogenicity Biosafety level Isolation	unknown 2 human feces	
MIGS-4	Geographic location	Senegal	IDA
MIGS-5	Sample collection time	September 2010	IDA
MIGS-4.1	Latitude	13.7167	IDA
MIGS-4.1	Longitude	-16.4167	IDA
MIGS-4.3	Depth	surface	IDA
MIGS-4.4	Altitude	51 m above sea level	IDA

Evidence codes - IDA: Inferred from Direct Assay; TAS: Traceable Author Statement (i.e., a direct report exists in the literature); NAS: Non-traceable Author Statement (i.e., not directly observed for the living, isolated sample, but based on a generally accepted property for the species, or anecdotal evidence). These evidence codes are from the Gene Ontology project [33]. If the evidence is IDA, then the property was directly observed for a live isolate by one of the authors or an expert mentioned in the acknowledgements.

**Figure 2:**
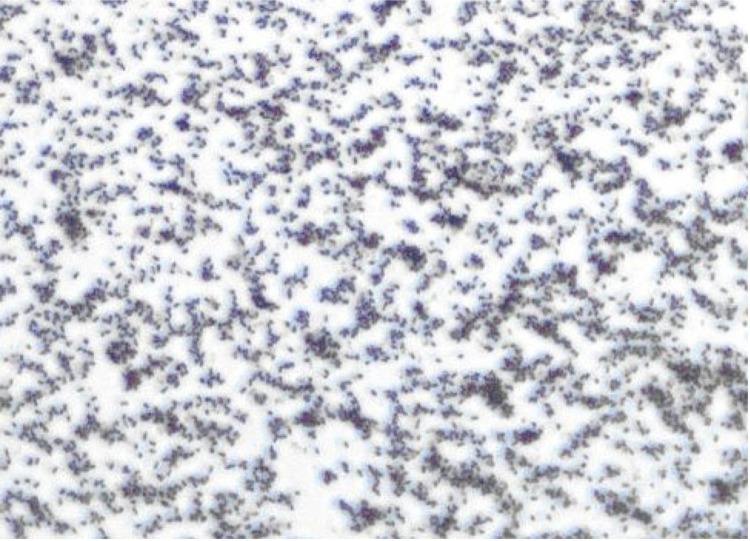
Gram staining of *T. senegalensis* strain JC301^T^

**Figure 3:**
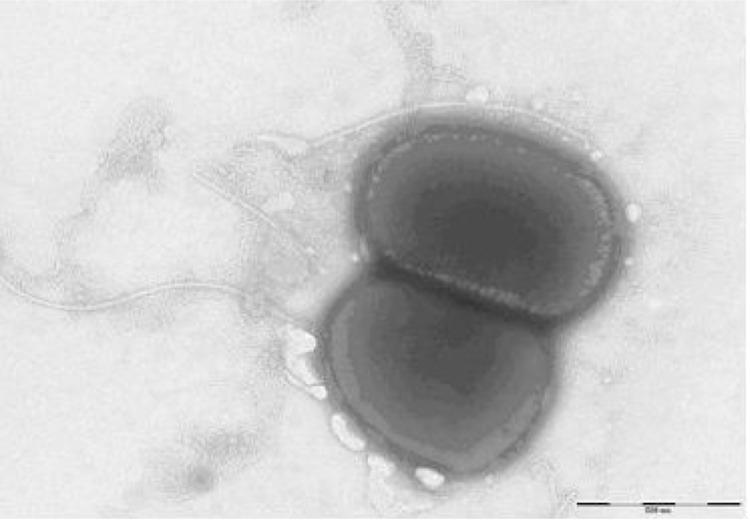
Transmission electron microscopy of *T. senegalensis* strain JC301^T^, using a Morgani 268D (Philips) at an operating voltage of 60kV. The scale bar represents 900 nm

Different growth temperatures (25, 30, 37, 45°C) were tested; growth occurred between 30 and 37°C, and optimal growth was observed at 37°C. Colonies were 1 mm in diameter on blood-enriched Columbia agar and Brain Heart Infusion (BHI) agar. Growth of the strain was tested in 5% sheep blood agar (BioMérieux), under anaerobic and microaerophilic conditions using GENbag anaer and GENbag microaer systems, respectively (BioMerieux), and under aerobic conditions, with or without 5% CO_2_. The strain grew optimally under aerobic conditions, however, weak growth was observed in microaerophilic and anaerobic atmospheres. Therefore, we concluded that strain JC301^T^ is a primarily aerobic, facultative anaerobic bacterium. The bacterial cells were Gram-positive, non-endospore-forming, short, irregular, motile rods (Figure 2), and had a mean diameter of 0.59 µm as determined using electron microscopy (Figure 3).

Strain JC301^T^ exhibited catalase but no oxidase activity. Using an API Rapid ID 32A system, positive reactions were obtained for urease, arginine dihydrolase, indole production, β-glucuronidase, mannose fermentation, alkaline phosphatase, arginine arylamidase, leucyl glycine arylamidase and histidine arylamidase. A weak positive reaction was obtained for pyroglutamyl arylamidase. Using API 50 CH assays, positive reactions were obtained for L-arabinose, D-galactose, D-glucose, D-maltose, D-saccharose, gentiobiose, arbutin, aesculin hydrolysis, and salicin. Nitrate reduction ability and β-galactosidase activities were found by using the API 20 NE kit. *T. senegalensis* strain JC301^T^ was susceptible to amoxicillin, imipenem, ciprofloxacin and gentamicin but resistant to trimethoprim/sulfamethoxazole and metronidazole. When compared with representative species from the suborder *Micrococcineae*, *T. senegalensis* gen. nov., sp. nov. strain JC301^T^ exhibited the phenotypic differences detailed in Table 2.

**Table 2:** Differential characteristics of *Timonella senegalensis* gen. nov., sp. nov., strain JC301^T^, *Cellulomonas fimi* strain ATCC484, *Oerskovia turbata* strain ATCC 25835 and *Sanguibacter keddieii* strain ST-74^T^

**Properties**	***T. senegalensis***	***C. fimi***	***O. turbata***	***S. keddieii***
Cell diameter (µm)	0.59	0.9 - 3.3	1.1	0.82
Oxygen requirement	facultatively anaerobic	aerobic	facultatively anaerobic	facultatively anaerobic
Colony color	white	yellow-white	yellow	yellow
Gram stain	+	+	+	+
Salt requirement	-	+	+	na
Motility	+	-	+	+
Cell Wall (major constituents)	alanine, glutamic acid and glucosamine	rhamnose, mannose, fucose and glucosamine	alanine, glutamic acid, glucosamine, muramic acid, galactose, lysine	alanine, glutamic acid, rhamnose and glucosamine
Endospore formation	-	-	-	-
**Production of**				
Phosphatase	+	na	-	na
Catalase	+	+	+	na
Oxidase	-	na	-	na
Nitrate reductase	+	-	+	-
Urease	-	-	-	na
β-galactosidase	+	+	+	-
N-acetyl-glucosamine	-	+	+	+
**Acid from**				
Inositol	-	+	-	-
Mannitol	-	-	-	-
Melibiose	-	-	-	+
Raffinose	-	-	-	+
Rhamnose	-	+	-	+
Sorbitol	-	na	+	+
**Utilization of**				
L-arabinose	+	-	+	+
D-ribose	-	+	+	-
D-glucose	+	+	+	+
D-fructose	-	-	+	+
D-mannose	-	+	+	+
D-maltose	+	+	+	+
D-lactose	-	-	+	+
Esculin	+	+	-	-
Habitat	human gut	soil	soil, decaying plant materials and clinical specimens	blood of healthy cow

na= data not available

**Figure 4:**
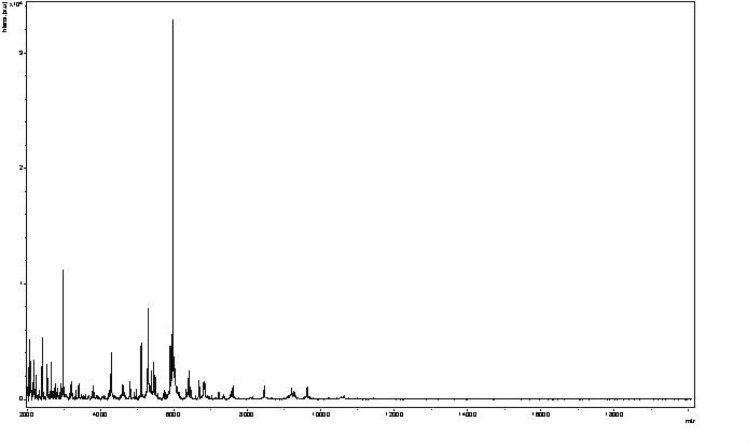
Reference mass spectrum from *T. senegalensis* strain JC301^T^. Spectra from 12 individual colonies were compared and a reference spectrum was generated.

**Figure 5:**
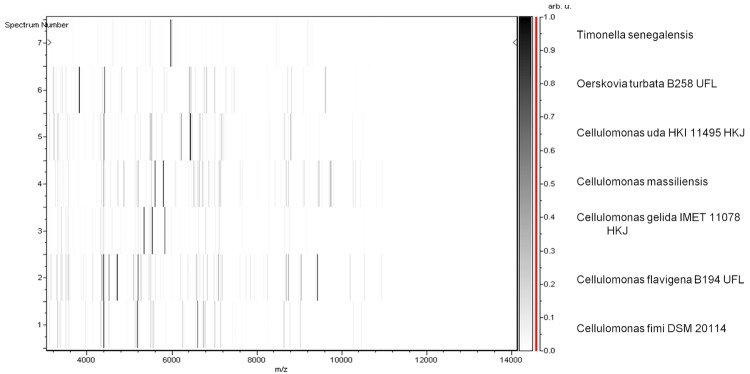
Gel view comparing *T. senegalensis* strain JC301^T^. The gel view displays the raw spectra of loaded spectrum files arranged in a pseudo-gel like look. The x-axis records the m/z value. The left y-axis displays the running spectrum number originating from subsequent spectra loading. The peak intensity is expressed by a Gray scale scheme code. The color bar and the right y-axis indicate the relation between the color a peak is displayed with and the peak intensity in arbitrary units. Displayed species are indicated on the left.

Matrix-assisted laser-desorption/ionization time-of-flight (MALDI-TOF) MS protein analysis was carried out as previously described [34]. Briefly, a pipette tip was used to pick one isolated bacterial colony from a culture agar plate and spread it as a thin film on a MTP 384 MALDI-TOF target plate (Bruker Daltonics, Germany). Twelve distinct deposits from twelve isolated colonies were performed for strain JC301^T^. Each smear was overlaid with 2 µl of matrix solution (a saturated solution of alpha-cyano-4-hydroxycinnamic acid) in 50% acetonitrile, 2.5% trifluoracetic acid and allowed to dry for 5 minutes. Next, measurements were taken with a Microflex spectrometer (Bruker). The spectra were recorded using a positive linear mode for the mass range of 2,000 to 20,000 Da (parameter settings: ion source 1 (ISI), 20kV; IS2, 18.5 kV; lens, 7 kV). A spectrum was obtained after 675 shots with variable laser power. The time of acquisition was between 30 seconds and 1 minute per spot. The twelve JC301^T^ spectra were imported into the MALDI Bio Typer software (version 2.0, Bruker) and analyzed via standard pattern matching (with default parameter settings) against the main spectra of 2,843 bacteria, including spectra from six validly published species of *Sanguibacter*, twenty-three validly published species of *Cellulomonas* and five validly published species of *Oerskovia* which were used as reference data. The method of identification included the m/z from 3,000 to 15,000 Da. For every spectrum, a maximum of 100 peaks at most were compared with the spectra in database. The resulting score enabled the identification of tested species: a score ≥ 2 with a validly publsihed species enabled identification at the species level, a score ≥ 1.7 but < 2 enabled identification at the genus level, and a score < 1.7 did not enable any identification. No significant MALDI-TOF score was obtained for strain JC301^T^ against the Bruker database, suggesting that our isolate was not a member of a known genus (Figures 4 and 5). We added the spectrum from strain JC301 ^T^ to our database.

## Genome sequencing information

### Genome project history

The organism was selected for sequencing on the basis of its phylogenetic position and 16S rRNA similarity to other members of the suborder *Micrococcineae*, and is part of a “culturomics” study of the human digestive flora aiming at isolating all bacterial species within human feces. It was first genome of *Timonella senegalensis* gen. nov., sp. nov. A summary of the project information is shown in Table 3. The Genbank accession number is CAHH00000000 and consists of 78 contigs. Table 3 shows the project information and its association with MIGS version 2.0 compliance [35].

**Table 3:** Project information

**MIGS ID**	**Property**	**Term**
MIGS-31	Finishing quality	High-quality draft
MIGS-28	Libraries used	Shot Gun, Paired-end 3 Kb library
		
MIGS-29	Sequencing platforms	454 GS FLX Titanium
MIGS-31.2	Fold coverage	22.05x
MIGS-30	Assemblers	Newbler version 2.5.3
MIGS-32	Gene calling method	Prodigal
	INSDC ID	PRJEA82329
	Genbank ID	CAHH00000000
	Genbank Date of Release	June 1, 2012
MIGS-13	Project relevance	Study of the human gut microbiome

### Growth conditions and DNA isolation

*T. senegalensis* sp. gen. nov. strain JC301^T^ (= CSUR P167 = DSMZ 25696) was grown aerobically on 5% sheep blood-enriched BHI agar at 37°C. The growth from four Petri dishes was collected and resuspended in 4×500 µl of TE buffer and stored at -20°C. Then, 500 µl of this suspension was thawed, centrifuged for 3 minutes at 10,000 rpm and resuspended in 4×100 µL of G2 buffer (EZ1 DNA Tissue Kit, Qiagen). An initial mechanical lysis was performed with glass powder and the Fastprep-24 device (Sample Preparation System, MP Biomedicals, USA) using two 20-seconds cycles. DNA was then treated with 2.5 µg/µL lysozyme for 30 minutes at 37°C and extracted using the BioRobot EZ1 Advanced XL (Qiagen). The DNA was then concentrated and purified using a QIAmp Kit (Qiagen). The yield and the concentration were measured at 754 ng/µL using the Quant-it Picogreen Kit (Invitrogen) on the Genios Tecan fluorometer.

### Genome sequencing and assembly

DNA (5 µg) was mechanically fragmented with a Hydroshear device (Digilab, Holliston, MA,USA) with an enrichment size of 3-4 kb. The DNA fragmentation was visualized using the Agilent 2100 BioAnalyzer on a DNA Labchip 7500 with an optimal size of 3.3 kb. The library was constructed using the 454 GS FLX Titanium paired-end protocol. Circularization and nebulization were performed and generated a pattern with an optimal size of 544 bp. After PCR amplification for 15 cycles and double size selection, the single-stranded paired-end library was then quantified using a Quant-it Ribogreen Kit (Invitrogen) using the Genios Tecan fluorometer. The library concentration equivalence was calculated as1.99× 10^9^ molecules/µL. The library was stored at -20°C until further use.

The shotgun library was clonally amplified with 0.5 cpb and the paired-end library was amplified with 1 cpb in four emPCR reactions using the GS Titanium SV emPCR Kit (Lib-L) v2 (Roche). The yields of the emPCR were 16.25% for the shotgun library and 15.92% for the paired-end library. These yields fall into the expected 5 to 20% range as per the Roche protocol.

Approximately 790,000 beads for a quarter region and 340,000 beads for an eighth region were loaded on the GS Titanium PicoTiterPlate PTP Kit 70×75 and sequenced with the GS FLX Titanium Sequencing Kit XLR70 (Roche). The run was performed overnight and then analyzed on a cluster using the gsRunBrowser and Newbler assembler (Roche). For the shotgun sequencing, 794,539 passed-filter wells were obtained. The sequencing generated 77.43 Mb with an average length of 190 bp. The passed-filter sequences were assembled using Newbler with 90% identity and 40 bp as overlap. The final assembly identified 78 contigs (between 1,468 bp and 199,104 bp) generated a genome size of 3.0 Mb, which corresponds to coverage of 22.05 genome equivalents.

### Genome annotation

Open Reading Frames (ORFs) were predicted using Prodigal [36] with default parameters but the predicted ORFs were excluded if they were spanning a sequencing gap region. The predicted bacterial protein sequences were searched against the GenBank database [37] and the Clusters of Orthologous Groups (COG) databases using BLASTP. The tRNAScanSE tool [38] was used to find tRNA genes, whereas ribosomal RNAs were found by using RNAmmer [39] and BLASTn against the GenBank database. Signal peptides and numbers of transmembrane helices were predicted using SignalP [40] and TMHMM [41] respectively. ORFans were identified if their BLASTP *E*-value was lower than 1e^-03^ for alignment length greater than 80 amino acids. If alignment lengths were smaller than 80 amino acids, we used an *E*-value of 1e-05. Such parameter thresholds have already been used in previous works to define ORFans. To estimate the mean level of nucleotide sequence similarity at the genome level between *T. senegalensis* strain JC301^T^, *Sanguibacter keddieii* strain ST-74^T^ (GenBank accession number CP001819) and *Cellulomonas fimi* strain ATCC 484 (GenBank accession number CP002666), we identified orthologous proteins using the Proteinortho software (version 1.4) and the following criteria: 30% amino acid identity and a *E*-value of 1e^-05^. The average percentages of nucleotide sequence identity between corresponding orthologous sets were then determined using the Needleman-Wunsch algorithm global alignment technique. Artemis [42] was used for data management and DNA Plotter [43] was used for visualization of genomic features. Mauve alignment tool was used for multiple genomic sequence alignment and visualization [44].

## Genome properties

**Figure 6:**
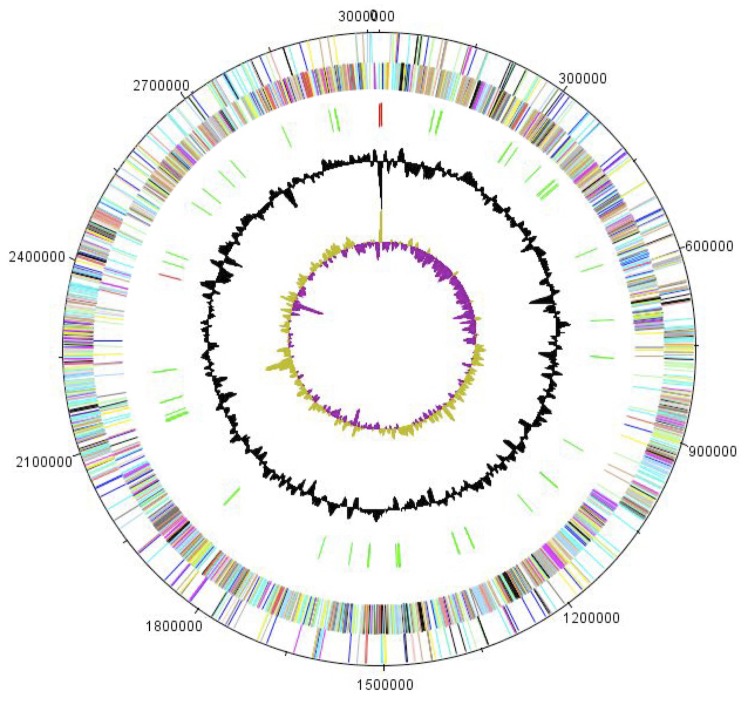
Graphical circular map of the chromosome. From the outside in, the outer two circles show open reading frames oriented in the forward (colored by COG categories) and reverse (colored by COG categories) direction, respectively. The third circle marks the rRNA gene operon (red) and tRNA genes (green). The fourth circle shows the G+C% content plot. The inner-most circle shows GC skew, purple indicating negative values whereas olive for positive values.

The genome of *T. senegalensis* sp. gen. nov. strain JC301^T^ is 3,010,102 bp long (1 chromosome, but no plasmid) with a 61.40% G+C content (Figure 6 and Table 4). Of the 2,793 predicted genes, 2,721 were protein-coding genes and 72 were RNAs. Six rRNA genes (two 16S rRNA, two 23S rRNA and two 5S rRNA) and 66 predicted tRNA genes were identified in the genome. A total of 1,949 genes (69.78%) were assigned a putative function. ORFans accounted for 285 (10.4%) of the genes. The remaining genes were annotated as hypothetical proteins. The distribution of genes into COGs functional categories is presented in Table 5. The properties and the statistics of the genome are summarized in Tables 4 and 5.

**Table 4:** Nucleotide content and gene count levels of the genome

**Attribute**	Value	% of total^a^
Genome size (bp)	3,010,102	
DNA coding region (bp)	2,656,410	88.24
DNA G+C content (bp)	1,848,203	61.40
Number of replicons	1	
Extrachromosomal elements	0	
Total genes	2,793	100
RNA genes	72	2.58
rRNA operons	2	
Protein-coding genes	2,721	97.42
Genes with function prediction	2,291	82.02
Genes assigned to COGs	1,949	69.78
Genes with peptide signals	153	5.48
Genes with transmembrane helices	659	23.60
CRISPR repeats	0	

^a^ The total is based on either the size of the genome in base pairs or the total number of protein coding genes in the annotated genome

**Table 5:** Number of genes associated with the 25 general COG functional categories

**Code**	**Value**	**%age**^a^	**Description**
J	149	5.47	Translation
A	1	0.03	RNA processing and modification
K	210	7.71	Transcription
L	122	4.48	Replication, recombination and repair
B	1	0.03	Chromatin structure and dynamics
D	18	0.66	Cell cycle control, mitosis and meiosis
Y	0	0	Nuclear structure
V	41	1.50	Defense mechanisms
T	90	3.30	Signal transduction mechanisms
M	102	3.75	Cell wall/membrane biogenesis
N	35	1.28	Cell motility
Z	0	0	Cytoskeleton
W	0	0	Extracellular structures
U	40	1.47	Intracellular trafficking and secretion
O	85	3.12	Posttranslational modification, protein turnover, chaperones
C	133	4.88	Energy production and conversion
G	249	9.15	Carbohydrate transport and metabolism
E	204	7.49	Amino acid transport and metabolism
F	68	2.50	Nucleotide transport and metabolism
H	75	2.75	Coenzyme transport and metabolism
I	64	2.35	Lipid transport and metabolism
P	140	5.14	Inorganic ion transport and metabolism
Q	37	1.36	Secondary metabolites biosynthesis, transport and catabolism
R	275	10.11	General function prediction only
S	152	5.58	Function unknown
-	772	28.37	Not in COGs

^a^ The total is based on the total number of protein coding genes in the annotated genome.

## Genome comparison with *Sanguibacter keddieii* and *Cellulomonas flavigena*

**Figure 7:**
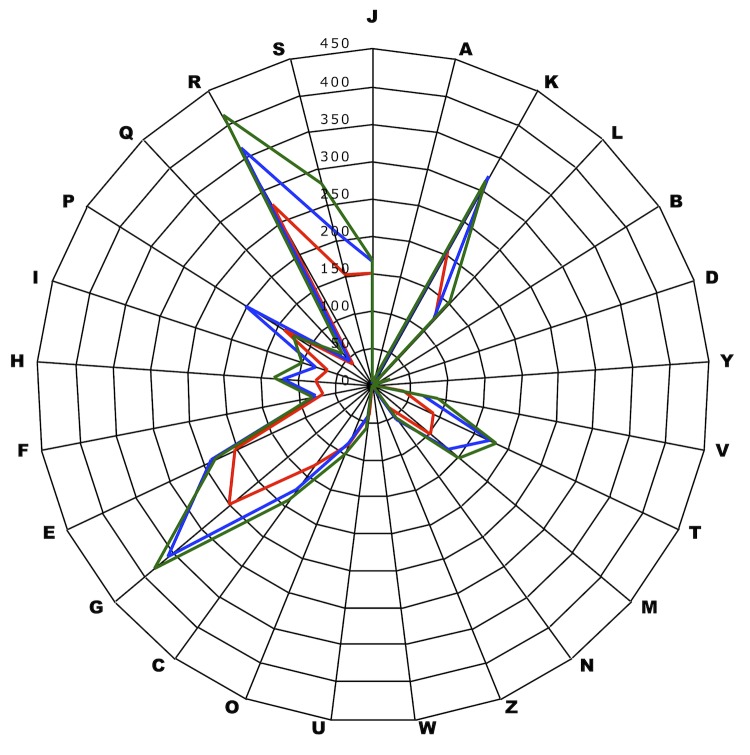
Distribution of functional classes of the predicted genes in the *T. senegalensis* strain JC301^T^ (colored in red), *S. keddieii* strain ST-74^T^ (colored in blue) and *C. fimi* strain ATCC 484 (colored in green) chromosomes according to the clusters of orthologous groups of proteins.

We compared the genome of T. senegalensis strain JC301^T^ with those of *Sanguibacter keddieii* strain ST-74^T^ (GenBank accession number CP001819) [45] and *Cellulomonas fimi* strain ATCC 484 ^T^ (GenBank accession number CP002666). The *T. senegalensis* genome is smaller in size than those of *S. keddieii* and *C. fimi* (3.0, 4.5 and 4.2 Mb, respectively). The G+C content of T. senegalensis is lower than that of *S. keddieii* and *C. fimi* (61.40%, 71.90% and 74.72%, respectively). The gene content of T. senegalensis is also lower than *S. keddieii* and *C. fimi* (2,793, 3,800 and 3,875 genes, respectively). Moreover, T. senegalensis presented a lower ratio of genes per Mb than *S. keddieii* and *C. fimi* (917, 931 and 922, respectively) and a comparable number of genes assigned to COGs (69.78%, 71.29% and 76.03%, respectively). However, the distribution of genes into COG categories (Figure 7) is not entirely similar in the three compared genomes. *T. senegalensis* sp. nov. exhibited a lower average genomic nucleotide sequence identity with S. keddiei (71.95%) and C. fimi (70.24%) than that observed between *S. keddiei* and *C. fimi* (76.94%). Table 6 summarizes the numbers of orthologous genes and the average percentage of nucleotide sequence identity between the different genomes studied.

**Table 6:** Numbers of orthologous protein shared between genomes (upper right triangle), average percentage similarity of nucleotides corresponding to orthologous protein shared between genomes (lower left triangle) and the numbers of proteins per genome (bold).

	*Timonella senegalensis*	*Sanguibacter keddieii*	*Cellulomonas fimi*
*Timonella senegalensis*	**2,721**	1,368	1,410
*Sanguibacter keddieii*	71.95	**3,710**	1,632
*Cellulomonas fimi*	70.24	76.94	**3,762**

## Carbohydrate metabolism

Experimentally, *T. senegalensis* strain JC301^T^ was able to grow under aerobic conditions and to utilize a variety of carbon substrates. Genome annotation clearly confirmed that this strain was able to use these carbon sources and to catabolize them via different pathways (glycolysis, pentose phosphate, TCA cycles and Entner-Doudoroff pathways). Strain JC301^T^ could effectively catabolize a variety of carbon substrates, such as D-fructose, L-arabinose, ribose, D-raffinose, xylose, D-galacturonate and D-glucuronate. Glycogen is a major intracellular carbon source reserve polymer. It is accumulated under conditions of limiting growth when an excess of carbon is available and other nutrients are deficient [46]. In strain JC301^T^, both the genes encoding the proteins required for glycogen biosynthesis and glycogen degradation are present, suggesting that it can survive for a longer period under carbohydrate starvation conditions. A variety of enzymes are present, including those required for gluconeogenesis and fermentation to lactate and acetate, as well as production of butyrate from branched amino acids. Acetyl-CoA can be used for anabolic purposes (fatty acid synthesis) or converted to acetate and butyrate. All four genes that encode enzymes for butyrate fermentation are found in the genome, including acetyl-CoA acetyltransferase, 3-hydroxybutyryl-CoA dehydrogenase, crotonase, and butyryl-CoA dehydrogenase.

### Fatty acid biosynthesis and oxidation

*T. senegalensis* strain JC301^T^ uses the non-mevalonate pathway for isoprenoid biosynthesis. All genes necessary for fatty acid and phospholipid biosynthesis are present. The strain also possesses the genes necessary for fatty acid biosynthesis initiation, keto group reduction, dehydration, and enoyl reduction. A cardiolipin synthetase gene is predicted in the JC301^T^ genome. This enzyme is found almost exclusively in certain bacterial membranes (plasma membrane and hydrogenosomes) and functions to generate an electrochemical potential for substrate transport and ATP synthesis [47]. In addition, the strain has genes of the fatty acid beta-oxidation system, suggesting that it can use fatty acids as carbon sources.

### Nucleotide metabolism

All genes required for *de novo* inosine monophosphate synthesis appear to be present in the *T. senegalensis* genome. Genes for uracil monophosphate synthesis are also organized in an operon interrupted by a conserved hypothetical ORF. The ORFs that encode the enzymes of uracil monophosphate (UMP) biosynthesis are closely related to the Gram-positive *S. keddieii*. Nucleoside monophosphate kinases for all types of nucleotides are present. Deoxyribonucleotides can be synthesized under both aerobic and anaerobic conditions by ribonucleoside-diphosphate and ribonucleoside-triphosphate reductases. Enzymes necessary for the purine and pyrimidine salvage pathway are also present. The purine salvage enzymes and uracil phosphoribosyltransferase are highly homologous to the corresponding enzymes of Gram-positive bacteria. Thymidine monophosphate is formed by thymidylate synthase from dUMP, providing the only interconversion pathway between pyrimidine nucleotides. In addition, there are four genes from the xanthine/uracil permease family of proteins involved in the transport of free bases. Thus, *T. senegalensis* can use exogenous bases and nucleosides. The *pst* operon encodes a phosphate-binding periplasmic protein, transport protein PstC, and a permease protein (PstA). All genes for phosphate lyase or other phosphonate degradation enzymes are present, suggesting that phosphates can be transported and further used.

### Respiration and proton transfer

All key proteins and protein complexes known to be important in aerobic complexes are present in the genome of *T. senegalensis*, including genes responsible for the synthesis of terminal cytochrome or quinol oxidases or complexes I, II, and III of the aerobic respiratory chain and ORFs for the synthesis of quinones or menaquinones. Two genes required for anaerobic respiration, arsenate reductase and ferredoxin reductase are present in the genome. Several proteins contributing to the proton gradient, including a proton:sodium-glutamate symporter, a sodium:proton antiporter, a V-type H^+^-translocating ATP synthase (EC 3.6.1.34), and a Na^+^-transporting ATP synthase (EC 3.6.3.15) are present in the genome.

### Transcription and translation

The *T. senegalensis* components of the transcriptional apparatus, consisting of the genes encoding the αββ′β" and ω subunits of RNA polymerase (RNAP), are similar to those of a Gram-positive polymerase. For transcription termination, one gene encodes a Rho factor similar to that of *S. keddieii*. In addition, homologs of NusA and NusB are also present. All the typical prokaryotic translation initiation factors, IF-1, IF-2, and IF-3, are present. Two ORFs for the elongation factor EF-G as well as EF-Tu, EF-Ts, and EF-p (elongation factor for peptide bond synthesis) genes are also present. *T. senegalensis* encodes three peptide chain release factors, RF-1, RF-2 and RF-3. Large and small ribosomal subunit proteins for the assembly of the ribosome are present in the genome. Modifying proteins such as ribosomal protein alanine acetyltransferase and large ribosomal subunit pseudouridine synthase subunits A, B, and D are present. Sixty-four ORFs code for tRNAs for all 20 amino acids. All types of tRNA ligases are present in the genome.

### Transporter system

Approximately 5% of the ORFs in the genome are dedicated to transport of a variety of compounds by primary and secondary transport systems. These transporters are energized by ATP, sodium, or proton gradients. There are 40 complete ABC transporter operons. The predominant substrates for ABC transporters appear to be oligopeptides and iron compounds. In contrast, there are three ABC transporters for amino acids. There are ABC transporters for other metal ions such as cobalt, nickel, manganese, zinc, and copper. The transmembrane sodium gradient appears to be as important for transport as the proton gradient. Most of the amino acid transporters are sodium-dependent. There are two potassium uptake systems: one is a sodium symporter, and the other is a proton symporter. Eight predicted sodium:proton antiporters are present in the genome. *T. senegalensis* uses these antiporters to balance ion gradients and to adjust to the pH changes in the gut environment. There are transporters for all of the essential ions and all the L-amino acids.

### Adaptability to human gut

Strain JC301^T^ was isolated from the human gut, suggesting that it can use substrates present in the colon. Accordingly, the complete pathway for gluconic acid degradation, including gluconate kinase and 6-phosphogluconate dehydrogenase was identified, in agreement with gluconate utilization.

The presence of stress-induced genes reflect the ability to cope with digestive (acid and bile) stresses. Regulation of intracellular pH is crucial for survival. Genome analysis of strain JC301^T^ revealed a complete *atp*BEFHAGDC operon, which is induced by acid and bile salts [48]. These stimuli also induce pyruvate-flavodoxin oxidoreductase and succinate dehydrogenase, involved in electron transport and ATP synthesis, as well as glutamate decarboxylase and aspartate ammonia-lyase, which regulates the homeostasis of intracellular pH [49]. Proteins involved in protection and repair of DNA are crucial for survival. Genome analysis demonstrated the presence of members of the SOS response including lexA, recA and uvrABC in *T. senegalensis* and *S. keddieii*. Moreover, the helix-destabilizing single-stranded DNA-binding protein (SSB), involved in DNA recombination and repair [50], as well as Dps (DNA-binding proteins from starved cells), which protects DNA against oxidative stress [51], are present in the genome. This reflects the ability to modulate envelope properties. In addition, strain JC301^T^ possesses an arsenal of genes for disulfide-reduction and elimination of reactive oxygen species, required for survival and activity within the gut against oxidative stress induced by bile. The occurrence of a sodium/bile acid symporter also reflects adaptation to the gut environment [52]. Moreover, genes encoding multidrug resistance transporters are present in *T. senegalensis* and *S. keddieii*, indicating an ability to cope with toxic compounds. The presence of two genes encoding heavy metal translocating P-type ATPases further suggests an adaptation to toxic environments. Thus, the genome content suggests *T. senegalensis* has significant environmental adaptation ability. Further genome analysis revealed the presence of several genes required for the inducibility of the different aspects of the chaperone and protease machinery. This suggests an ability to efficiently and rapidly adapt to stressful environments, such as would be found in a human host.

## Conclusion

### Description of *Timonella gen. nov.*

*Timonella* (Ti.mon.el′la N.L. fem. N. Timonella, from Timone, the name of the hospital in Marseille where strain JC301^T^ was first cultivated).

Gram-positive rods. Facultatively anaerobic. Mesophilic. Motile. catalase-positive. Absent oxidase. Positive for urease, arginine dihydrolase, indole production, β-glucuronidase, mannose fermentation, alkaline phosphatase alcaline, arginine arylamidase, leucyl glycine arylamidase and histidine arylamidase. Habitat: human digestive tract. Type species: *Timonella senegalensis*.

### Description of *Timonella senegalensis* gen. nov., sp. nov.

*Timonella senegalensis* (se.ne.gal.e′n.sis. L. gen. masc. n. *senegalensis*, pertaining to Senegal, the country from which the patient came).

Gram-positive, catalase-positive, oxidase-negative and facultatively anaerobic. Cells are irregular, non-endospore forming, short, irregular motile rods with a mean diameter of 0.59 µm. Colonies are white, circular and convex with entire edges on 5% sheep blood agar in aerobic atmosphere at 37°C. Diffusible pigments are not produced. Optimal growth occurs under aerobic conditions. Grows at 30-37 °C (optimum 37 °C). Cells are positive for urease, arginine dihydrolase, indole production, β-glucuronidase, mannose fermentation, alkaline phosphatase alcaline, arginine arylamidase, leucyl glycine arylamidase and histidine arylamidase (API50CH). Cells have nitrate reduction ability and β-galactosidase activity (API 20 NE kit). Positive reactions for L-arabinose, D-galactose, D-glucose, D-maltose, D-saccharose, gentiobiose, arbutine, esculine, salicine (API 50 CH). A weak reaction was obtained for pyroglutamyl arylamidase. Susceptible to amoxicillin, imipenem, ciprofloxacin and gentamicin but resistant to trimethoprim/sulfamethoxazole and metronidazole. The potential pathogenesis of the type strain JC301^T^ is unknown.

The type strain is JC301^T^ (= CSUR P167 = DSMZ 25696) was isolated from the fecal flora of a healthy patient from Dielmo (a rural village in the Guinean-Sudanian zone in Senegal). The genomic DNA G+C content of the type strain is 61.4 mol%. The 16S rRNA gene sequences were deposited in GenBank with the accession number JN657220. The whole-genome shotgun sequence of *T. senegalensis* strain JC301^T^ has been deposited in GenBank/DDBJ/EMBL under accession number CAHH00000000.
